# Supplementary material for: A UMLS-based spell checker for natural language processing in vaccine safety
Source: BMC Med Inform Decis Mak. 2007 Feb 12;7:3. doi: 10.1186/1472-6947-7-3 (PMC1805499; doi:10.1186/1472-6947-7-3)
Supplement: Additional file 13 — RAP application directory. Sets up directory for RAP (RDF application for PHP) source code [file 1472-6947-7-3-S13.gz › rap/doc/tests/test_NamedGraphsMem.htm]

### RDF API for PHP V0.9.1

# Test: Using the Dataset API

  
This example is part of the RAP - Rdf API for PHP documentation.

Working with NamedGraphSets:

<?php   
// Include RAP   
define("RDFAPI\_INCLUDE\_DIR", "./../../api/");   
include(RDFAPI\_INCLUDE\_DIR . "RdfAPI.php");

//get a GraphSet from the ModelFactory   
$graphset = ModelFactory::getDatasetMem('Dataset1');

// Create a new NamedGraphsMem   
$graph1 = new NamedGraphMem('http://graph1');   
$graph2 =& $graphset->createGraph('http://graph2');

//create Quads   
$quad1= new Quad(new Resource('http://graph3'),new Resource('http://subject1'),new Resource('http://predicate1'),new Resource('http://object1'));   
$quad2= new Quad(new Resource('http://graph3'),new Resource('http://subject2'),new Resource('http://predicate2'),new Resource('http://object2'));   
$quad3= new Quad(new Resource('http://graph4'),new Resource('http://subject3'),new Resource('http://predicate3'),new Resource('http://object3'));   
$quad5= new Quad(new Resource('http://graph4'),new Resource('http://subject5'),new Resource('http://predicate5'),new Resource('http://object5'));

//add Quads to the GraphSet. Thee needed NamedGraphs will be created automatically   
$graphset->addQuad($quad1);   
$graphset->addQuad($quad3);   
$graphset->addQuad($quad2);

//add a NamedGraph to the Set   
$graphset->addNamedGraph($graph1);

//Is the Graph "http://graph1" part of the GraphSet?   
echo '<b>Is the Graph "http://graph1" part of the GraphSet?</b><BR>';   
var\_dump($graphset->containsNamedGraph('http://graph1'));   
echo'<p/>';

//remove 'http://graph2' from the set   
$graphset->removeNamedGraph('http://graph2');

//Is the Graph "http://graph2" part of the GraphSet?   
echo '<b>Is the Graph "http://graph2" part of the GraphSet?</b><BR>';   
var\_dump($graphset->containsNamedGraph('http://graph2'));   
echo'<p/>';

//creat basic statement   
$statement=new Statement(new Resource('http://subject4'),new Resource('http://predicate4'),new Resource('http://object4'));

//get a Graph from the Set and add a basic statement   
$graph4=&$graphset->getNamedGraph('http://graph4');   
$graph4->add($statement);

//remove Quad5   
$graphset->removeQuad($quad5);

//get an iterator over all Quads   
echo '<p/><b>All Quads:</b><BR>';   
for($iterator = $graphset->findInNamedGraphs(null,null,null,null); $iterator->valid(); $iterator->next())   
{   
  $quad=$iterator->current();   
  echo $quad->toString().'<BR>';   
};

//get an iterator over all Quads in 'http://graph3'   
echo '<p/><b>All Quads in http://graph3:</b><BR>';   
for($iterator = $graphset->findInNamedGraphs(new resource('http://graph3'),null,null,null); $iterator->valid(); $iterator->next())   
{   
  $quad=$iterator->current();   
  echo $quad->toString().'<BR>';   
};

//how many Quads are in the whole GraphSet   
echo '<p/><b>How many Quads are in the whole GraphSet?</b><BR>';   
echo $graphset->countQuads();

//does the GraphSet contain the Quad (http://graph4, http://subject4, http://predicate4, http://object4) ?   
echo '<p/><b>Does the GraphSet contain the Quad (http://graph4, http://subject4, http://predicate4, http://object4) ?</b><BR>';   
var\_dump($graphset->containsQuad(new Resource('http://graph4'),new Resource('http://subject4'),new Resource('http://predicate4'),new Resource('http://object4')));   
?>

Output:

**Is the Graph "http://graph1" part of the GraphSet?**   
bool(true)

**Is the Graph "http://graph2" part of the GraphSet?**   
bool(false)

**All Quads:**   
GraphName(http://graph3) Triple(Resource("http://subject1"), Resource("http://predicate1"), Resource("http://object1"))   
GraphName(http://graph3) Triple(Resource("http://subject2"), Resource("http://predicate2"), Resource("http://object2"))   
GraphName(http://graph4) Triple(Resource("http://subject3"), Resource("http://predicate3"), Resource("http://object3"))   
GraphName(http://graph4) Triple(Resource("http://subject4"), Resource("http://predicate4"), Resource("http://object4"))

**All Quads in http://graph3:**   
GraphName(http://graph3) Triple(Resource("http://subject1"), Resource("http://predicate1"), Resource("http://object1"))   
GraphName(http://graph3) Triple(Resource("http://subject2"), Resource("http://predicate2"), Resource("http://object2"))

**How many Quads are in the whole GraphSet?**   
4

**Does the GraphSet contain the Quad (http://graph4, http://subject4, http://predicate4, http://object4) ?**   
bool(true)
